# HIV exposure and related newborn morbidity and mortality in the University Teaching Hospital of Yaoundé, Cameroon

**DOI:** 10.4314/pamj.v8i1.71160

**Published:** 2011-04-16

**Authors:** Francisca Monebenimp, Dorothee Ella Nga-Essono, Anne-Cecile Zoung-Kany Bissek, David Chelo, Ekoe Tetanye

**Affiliations:** 1 Department of Pediatrics, Faculty of Medicine and Biomedical Sciences, University of Yaounde I, Cameroon; 2 Neonatology Unit, University Teaching hospital of Yaounde, Cameroon; 3 Mother and Child Centre of the Chantal BIYA Foundation Yaounde, Cameroon

**Keywords:** HIV exposure, newborn, morbidity, mortality, Cameroon

## Abstract

**Introduction:**

Few studies have established the role of maternal HIV infection on neonatal disease and death. In order to determine whether neonatal morbidity and mortality were associated to maternal HIV infection, a case-control study was conducted in the neonatal unit of the University Teaching Hospital of Yaoundé from July 2006 to December 2007.

**Methods:**

Babies born from HIV positive mothers were recruited as cases. For each case, two babies born from HIV negative mothers were selected as controls. Informed verbal consent was obtained from the mother before inclusion of the newborn in the study. Information on demographics, history of pregnancy, diseases and outcome of the newborns were extracted from patients’ files. The distribution of these parameters between cases and control was analyzed using chi-square. Association of demographics, clinical and paraclinical parameters with mortality was explored using univariate analysis and logistic regression. Data were analyzed using Epi Info version 3.5.1 Windows.

**Results:**

Out of 240 newborns enrolled, 80 were cases and were 160 controls. The mean age of cases was 1.69±2.73 days compared to 1.46±2.36 days for controls (p=0.26). Cases significantly differed from controls on mother’s marital status (p=0.02), level of education (p<0.001), number of prenatal consultations (p<0.001), anemia chemoprophylaxis (p=0.01) and drug abuse (p<0.001). Cases and controls were similar for prematurity, respiratory distress, sepsis, meningitis and urinary tract infection. The death rate was identical in both groups (p=0.52). Using Univariate analysis, risk factors associated to mortality in both groups were prematurity (p<0.001) and low birth weight (p<0.001).

**Conclusion:**

This study showed no statistical difference in morbidity and mortality between newborns from HIV positive and HIV negative mothers.

## Introduction

In Africa, HIV infection rates among pregnant women ranged from 15 to 40 percent in countries with the highest overall HIV prevalence in 2006

[1]. Even though HIV is not a direct cause of neonatal death, maternal HIV status affects newborn survival by causing an increased risk of stillbirth and death in the neonatal period and infancy; this increase risk extend to those babies who do not become HIV positive [2]. The interaction of HIV with other infections and the indirect effects of HIV, such as poverty and maternal illness, contribute to the poor outcome of these newborns. It has been established that newborns exposed to mother’s HIV infection have a higher mortality rate than those not exposed [3-5]. HIV is more prevalent in Cameroon, and the pattern of HIV epidemic is changing overtime with a prevalence in the general population that went from 5.5 in 2001 to 5.3 in 2009 [2]. Women attending antenatal visits are more aware of being tested for HIV infection. The rate of HIV testing acceptance during pregnancy went from 31% in 2005, 77.7% in 2007 to 83.4% in 2009 [6]. To answer the question whether maternal HIV infection is associated to newborn’s morbidity and mortality in our setting, we performed a hospital based study, with the aim of addressing the issue of building a comprehensive communicative approach in antenatal care (ANC) clinic for the prevention of mother-to-child transmission of HIV.

## Methods

This was a case control study carried out at the neonatology Unit of the University Teaching Hospital of Yaoundé (UTHY) from July 2006 to December 2007. The UTHY is a referral hospital that had a 5 bedded labor room. The staff of the maternity comprises 7 gynecologists /obstetricians, 9 residents and 18 nurses including 2 midwives. Cases included in the study were newborns from mothers with known HIV positive serology, born in the maternity of the UTHY and admitted in the neonatal unit of the same hospital. Newborns from HIV negative mothers, born in the same hospital and admitted in the same neonatal unit were taken as controls. Newborns with life threatening congenital anomalies were excluded from the study because of the potential interaction with morbidity and mortality.

Since 2005, the hospital policy to prevent mother-to-child transmission of HIV is to admit in the neonatal unit all baby from HIV positive mothers, free of charges, for up to 72 hours. The aim of this policy is to insure that mothers understand the administration of antiretroviral drugs and the feeding option of the newborn while assuring psychosocial support to the parents. The package for HIV positive pregnant women included antiretroviral (ARV) drugs such as the combined tablets of Zidovudine and Lamivudine at 300/150 mg every 12 hours starting at 28 weeks of pregnancy and a tablet of Nevirapine 200 mg at onset of labor. Newborns receive Zidovudine 4 mg/kg/day every 12 hours for 4 weeks and a single dose of Nevirapine 2 mg/kg within 72 hours of delivery.

Informed verbal consent was obtained from mothers before enrolment in the study. Ethical clearance was obtained from the National Ethical Committee.

The selection of cases was as followed: babies born from an HIV positive mother were randomly selected from the neonatology patient register. In order to increase the power of the study, for each case, two babies born from HIV negative mothers were selected as controls. This was done until fulfillment of the calculated sample size, taking into account a proportion of 15% of HIV infected newborns.

Information was extracted from mothers’ files regarding history of pregnancy and delivery and other relevant demographics. Information collected from the newborn files included age and sex of the baby, anthropometric parameters, glycemia, calcemia, infection and other clinical problems presented during the length of stay in hospital. Neonatal sepsis, meningitis and urinary tract infection were validated when bacteria were isolated respectively in the culture of blood, central nervous system fluid and urine. Data were analyzed using Epi Info version 3.5.1 Windows. No matching criterion was used in the analysis. The Chi square test was performed to compare proportions and the level of significance was set at 0.05. Variables significantly associated with mortality at a level of p<0.07 on the univariate analysis and those variables that we thought could be associated with mortality in a fishing expedition were further put in a logistic regression model to identify independent risk factors. The assumption was made for a protective effect of malaria chemoprophylaxis.

## Results

A total of 240 newborns were enrolled in the study. A male newborn with spina bifida and a female with omphalocele were excluded from the study. Out of the 80 newborns from HIV-positive mothers, 42 (52.5%) were males and 38 (47.5%) females. In the group of newborns from HIV-negative mothers, 85 (53.1%) were males and 75 (46.9%) females (p=0.07). The age range of the study population varied from birth to 13 days. The more represented age range was from 2 to 4 days, 28 (35%) cases compared to 77 (48.1%) controls. The mean age in the case group was 2.13±3.34 days compared to 1.46±2.36 days for controls (p=0.26).

Table 1 shows the demographic features and antenatal characteristics of HIV-positive and HIV-negative mothers. HIV positive mothers, 46 (57.5%) were more likely to attend UTHY ANC than HIV negative mothers (n=70, 43.8%, p=0.03). The mean maternal ages between HIV positive and HIV negative mothers was comparable, 27.97±4.86 years in cases and 26.99±5.34 years in controls (p=0.10). Most of the mothers in the case group were single 47 (58.8%) and multiparous 60 (75%) while in the control group most of them were respectively married 89 (56.6%) (p=0.02) and 101 (63.1%) were multiparous (p=0.39). As far as education was concerned, the majority of mothers of the study population was at college level, 48 (60%) for cases and 118 (73.8%) for controls (p<0.001). Drug abuse was reported by 6 mothers in the HIV positive group compared to 1 mother in the HIV negative group (p<0.001). Alcohol was reported by 4 and heavy tobacco smoking by 2 HIV positive mothers; one HIV negative mother reported alcohol consumption. Malaria chemoprophylaxis during pregnancy was noted in 71 cases (88.8%) compared to 148 controls (93.1%) (p<0.18) but for anemia chemoprophylaxis 73 (91.2%) of HIV positive mothers received ion and folate tablets against 157 (98.1%) in the group of HIV negative mothers (p=0.01). Malaria in pregnancy was observed in 19 (23.8 %) cases and 17 (10.6%) controls (p<0.001). HIV-positive women who received antiretroviral therapy on the basis of their lymphocytes count were 74 (92.5%) in number and 11 (14.8%) were put on tritherapy throughout pregnancy; 36 (48.6%) were on Zidovudine during pregnancy plus Nevirapine at onset of labor while 17 (22.9%) had Combivir without Nevirapine at onset of labor and 10 (13.5%) had Nevirapine alone at onset of labor.

HIV testing was done and obtained at labor for 6 HIV positive mothers. These women did not receive ARV but their offspring had Nevirapine and Zidovudine as the other newborns. Looking at t history of delivery, premature rupture of membranes was noticed in 25 cases (31.3%) compared to 75 (46.9%) controls (p=0.01). Most of the newborns were delivered vaginally, 63 (78.8%) in HIV exposed babies and 115 (71.9%) in newborns from HIV negative mothers. The difference was not statistically significant (p=0.13). Premature deliveries were 22 (27.5%) in cases compared to 34 (21.9%) in controls (p=0.41). The mean weight in HIV exposed newborns was 2950±579g while it was 2969±748g for controls (p=0.9). The mean height was 48.43±2.66 cm for cases compared to 48.92±3.76 cm for controls (= 0.14).

Table 2 illustrates morbidity and mortality profile of the newborns from HIV positive and HIV negative mothers. The number of newborns in the case group with an Apgar score below 6 at 5 minutes was 14 (17%) compared to 53 (33.1%) in the control group p<0.001). There was no statistically significant difference between the newborns from HIV positive mothers and those from HIV negative mothers as concerns low birth weight (p=0.53), body temperature (p= 0.30), metabolic disorders such as hypoglycemia (p=039) and hypocalcemia (p=0.77) and neonatal infection, septicemia (p=053), meningitis (p=0.44), urinary tract infection (p=031). Among the cases, 14 (17.5%) were on breastfeeding while 154 (96.3%) controls were on the same choice of feeding (p<0.001). The length of stay in hospital was comparable in both groups 9.1± 8.3 days for cases against 8.8± 8.6 days for controls (p=0.57).

The number of death in cases was 8 (10%) compared to 15 (9.4%) in controls and the difference between the two groups was not statistically significant (p=0.52). The univariate analysis for mortality in table 3 shows that factors associated with mortality were prematurity (OR =3.6; 95% CI =1.47-8.87; p=0.006) and low birth weight (OR=3.31; 95% CI = 1.3-8.09; p=0.009). None of the covariates studied was associated with neonatal mortality in the logistic regression model performed.

## Discussion

The study sample represented (8.3%) of the 956 HIV exposed and (7.6%) of the 2103 non-HIV exposed newborns delivered in the UTH maternity from July 2006 to December 2007. In this study, HIV positive mothers had a low level of education and were more likely to be single compared to HIV negative mothers as shown in Zambia and Rwanda [7, 9,10]. The two groups of mothers were comparable as regards to age and income generating activities as shown in a previous study in Cameroon [11].

**Prenatal and delivery history**

The majority of HIV positive and HIV negative mothers fulfilled the World Health Organization requirements of at least 4 ANC during pregnancy [12,13]. The number of antenatal consultations was similar in both groups of mothers; though a higher proportion of HIV positive mothers had on average 4 ANC visits more than HIV negative mothers. In addition, HIV positive mothers were more likely to come to ANC visits at the UTHY probably because of their related medical problems, as observed with the higher proportion of malaria in pregnancy. This disease was highly noticed in a study in Malawi [14] where the prevalence of malaria in HIV positive mothers reached (56.3%) compared to (36.5%) for HIV negative mothers. The discrepancy could be explained by the fact that our study population was urban in majority; in addition, a high proportion of women received malaria prophylaxis (88.8% of HIV positive mothers and 92.5 % of the HIV negative mothers). Iron deficiency anemia is the most common cause of anemia in pregnancy. The rate of malaria prophylaxis in this study was considerable and the majority of women had the required number of ANC visits; we can therefore presume that these women received nutritional counseling, iron and folic acid for anemia prophylaxis. Theses interventions are important in reducing anemia in pregnancy [13] and might explain why we did not find a single case of anemia in our study.

**Neonates morbidity and mortality**

The majority of newborns were males in the two groups but the difference was not statistically significant as pointed out by some authors [15,16]. The prevalence of prematurity was high in the two groups and the difference was not statistically significant. This was in accordance with the findings of Lepage et al [7]. Temmerman et al noticed a significant difference, 21.1% in the group of HIV infected mothers compared to 9.4% in HIV negative mothers [2] but, in this study, mothers were recruited as cases on the basis of having a low birth weight that could bias the result towards prematurity. Further, Haeri et al found that prematurity was more common among HIV-positive women than uninfected women (OR =2.27, 95% CI= 1.22-4.25) [17] but mothers were more likely to have heavy tobacco smoking and cocaine use. Neonatal asphyxia was high among cases compared to controls. This finding could be explained by the fact that the controls were newborns hospitalized in the neonatal unit because of birth trauma as this was a major reason for admission. The number of low birth weights was not statistically different between neonates born from HIV positive and HIV negative mothers. This finding does not concord with Ezeaka et al in Nigeria [18] who found that there were three times more low birth weights in the HIV positive mothers than in the HIV uninfected mothers (OR=3.47; 95 CI=1.69-7.27; P= 0.0003). The mean weight of newborns was similar in both groups. This was contrary to other studies which found that newborns exposed to HIV were more likely to have low birth weights [2,7]. Considering the adequate numbers of antenatal care we could assume that infected women were well managed during pregnancy, and the fact that they took antiretroviral therapy early enough, before the third trimester of pregnancy, could explain why the difference of mean weight was not statistically significant. These findings are similar to those of other authors who showed that anthropometric measurements of infants born from asymptomatic HIV infected mothers are comparable to those in the general population in contrast to children of drug-addicted mothers [19] in our study, there was only one HIV negative mother that was drug-addicted. Neonatal mortality was similar in the two groups, as found in other studies in Rwanda, Malawi, Zambia and Tanzania [7,8] . Neonatal death rates were 94 per 1000 in HIV exposed babies compared to 100 per 1000 on controls. These figures were high compared to the 40 per 1000 reported at national level [20]. The explanation could reside on the fact that this is a referral hospital and many of the deliveries were conducted in women who were not managed during pregnancy in this hospital and who came late, the high level of birth asphyxia found being part of this delay.

Factors associated with mortality in the univariate analysis were prematurity and low birth weight even though these and other variables analyzed did not appear as risk factors for mortality in a logistic regression model. However, some authors have shown that prematurity and low birth weight were considerably associated to morbidity and mortality in the neonatal period as well as in the first year of life [21-23] and that there was a direct correlation between vertical transmission of HIV, low birth weight and high mortality [23-26].

This study has a limitation that it could not tell strongly the number of mothers on ARV therapy that was symptomatic and their biological profiles. So there is a need to address this issue in further studies as misclassification bias might lead to the failure to pick up a difference, if any, between the two groups of neonates as far as prematurity, low birth weight and mean birth weight are concerned. Taking into consideration the variables that showed a trend to significance, the sample size was small enough to pick up differences between newborns from HIV positive and HIV negative mothers. A bias could have been introduced because of the choice of controls as they were more likely to have neonatal asphyxia that was the primary reason for admission in the neonatal unit. This was a hospital based study which did not cover the overall neonatal period; therefore these results could not be generalized to the community.

## Conclusion

This case control study showed that mortality rate was similar in newborns from HIV positive mothers and HIV negative mothers. Prematurity and low birth weight were potential risk factors for mortality in the univariate analysis but failed to be demonstrative in a logistic regression model. The impact of maternal HIV infection might not be evident in the neonatal period. Further studies are in progress and will take into account HIV positive mothers’ clinical and biological stages. However, health professionals should be aware of and properly address the issue of prematurity and low birth weight when taking care of HIV-positive pregnant women.

## Competing interests

The authors declare no competing interests.

## Authors’ contribution

ICMJE authorship criteria met. All the authors have read and approve the final version of the manuscript.

## Acknowledgements

Acknowledgments addressed to the neonatal and maternity staff of the University teaching Hospital of Yaoundé for their collaboration in conducting this study.

## Figures and Tables

**Table 1:** Sociodemographic and Antenatal characteristics of HIV- positive and HIV-negative mothers of the newborns, University Teaching Hospital, July 2006-December 2007

Variable	HIV+ mothers	HIV-mothers	P value
	n=80	n=160	
**Demographics**			
Mean age of the mothers (years)	27.32 ± 5.20	26.99 ± 5.34	0.10
Married mothers (n %)	33 (41.3)	89 (55.6)	0.02
College level of education (n %)	48 (60)	118 (73.8)	<0.001
Income activity generating (n %)	40 (50)	63 (39.4)	0.07
**History of pregnancy and delivery**			
Multiparity (n %)	64 (80)	120 (75)	0.39
Drug abuse (n %)	6 (7.5)	1 (0.6)	<0.001
UTHY site of ANC	46 (57.5)	70 (43.8)	0.03
At least 4 ANC (n %)	70 (87.5)	117 (73.1)	<0.001
Malaria in pregnancy (n %)	19 (23.8)	17 (10.6)	<0.001
Anemia in pregnancy (n %)	0	0	
Syphilis (n %)	1 (1.3)	3 (1.9)	0.58
Malaria chemoprophylaxis (n %)	71 (88.8)	148 (92.5)	0.18
Anemia chemoprophylaxis (n %)	73 (91.3)	157 (98.1)	0.01
Gestational age ≤ 37 weeks (n %)	22 (27.5)	35 (21.9)	0.41
Normal delivery (n %)	63 (78.8)	115 (71.9	0.13
Premature rupture of membranes (n %)	25 (31.3)	75 (46.9)	0.01
Choice of breastfeeding	14 (17.5)	154 (96.3)	<0.001

UTHY: University Teaching Hospital of Yaoundé

**Table 2:** Morbidity and mortality profile of the neonates born from HIV-positive mothers and HIV-negative mothers, University Teaching Hospital, July 2006-December 2007

variable	Newborns from HIV+	Newborns from	P value
	mothers	HIV-mothers	
	n=80	n=160	
Neonatal asphyxia (n %)	14 (17.5)	53 (33.1)	<0.001
Low birth weight (n %)	16 (20)	35 (21.8)	0.53
Mean birth weight (g)	2950±578	2969±748	0.90
Mean height (cm)	48.43±2.66	48.92±3.76	0.14
Hypothermia (n %)	23 (28.8)	61 (38.1)	0.30
Hypoglycemia (n %)	10 (12.5))	17 (10.6)	0.39
Hypocalcaemia (n %)	9 (11.3)	20 (12.5)	0.77
Neonatal sepsis (n %)	9 (11.3)	19 (11.9)	0.53
Neonatal meningitis (n %)	4 (5.0)	6 (3.8)	0.44
Neonatal respiratory distress (n %)	12 (15)	20 (12.5)	0.36
Urinary tract infection (n %)	3 (3.8)	3 (1.9)	0.31
Neonatal jaundice (n %)	21 (26.3)	42 (26.3)	0.55
Mean length of stay in hospital (days)	9.1 ± 8.3	8.8 ± 8.6	0.57
Neonatal death (n %)	8 (10)	15 (9.4)	0.52

**Table 3:** Univariate analysis and logistic regression of risk factors for mortality in neonates born from HIV-positive and HIV-negative mothers, University Teaching Hospital, July 2006-December 2007

Variables	Newborns from HIV+ mothers n(%)	Newborns from HIV-mothers n(%)	OR	CI (95%)	P value
**Univariate analysis**					
Income generating activity	4 (10)	6 (9.5)	1.02	0.43-2.44	0.48
Malaria in pregnancy	1 (5.3)	1 (5.9)	0.51	0.11-2.28	0.28
At least 4 ANC	7 (10)	10 (8.5)	1.30	0.47-3.56	0.38
Malaria chemoprophylaxis	8 (11.3)	12 (8.1)	0.58	0.16-2.12	0.30
Premature rupture of membranes	2 (8.0)	7 (9.3)	0.89	0.36-2.19	0.49
Prematurity	4 (25)	6 (18.8)	3.6	1.47-8.87	0.006
Low birth weight < 2500g	3 (18.8)	7 (20)	3.31	1.3-8.09	0.009
Neonatal sepsis	0 (0)	2 (10.5)	0.70	0.15-3.15	0.48
Neonatal meningitis	1 (25)	0 (0)	1.04	0.12-8.6	0.64
Neonatal respiratory distress	4 (33.3)	6 (30)	2.76	0.51-14.82	0.20
Anemia	0 (0)	2 (40)	2.38	0.4-11.70	0.25
**Multivariate analysis**					
Prematurity	-	-	5.69	(0.74-43.54)	0.09
Low birth weight	-	-	2.54	(0.33-19.34)	0.36
Malaria chemoprophylaxis	-	-	3.92	(0.77-19.79)	0.09
Premature rupture of membranes	-	-	0.88	(0.25-3.06)	0.84
Malaria in pregnancy	-	-	0.65	(0.07-5.66)	0.70
At least 4 ANC	-	-	1.06	(0.30-3.75)	0.91
